# Muscle Function Impairment in Crohn’s Disease Patients: Risk Factors and Clinical Implications—Single-Tertiary-Center Experience

**DOI:** 10.3390/life16050790

**Published:** 2026-05-08

**Authors:** Jelena Spiric Milovancevic, Aleksandar Toplicanin, Sasa Vuksanovic, Srdjan Djuranovic, Aleksandra Pavlovic Markovic, Sanja Mazic, Marina Djelic Blagojevic, Aleksandra Sokic-Milutinovic

**Affiliations:** 1 Clinic for Gastroenterohepatology, University Clinical Centre of Serbia, Koste Todorovica 2, 11000 Belgrade, Serbia; aleksandartoplicanin4@gmail.com (A.T.); sashavukasanovic9@gmail.com (S.V.); aleksandra.sokic.milutinovic@med.bg.ac.rs (A.S.-M.); 2 School of Medicine, University of Belgrade, 11000 Belgrade, Serbiamarina.djelic@med.bg.ac.rs (M.D.B.); 3 Institute of Medical Physiology “Richard Burian”, University of Belgrade, 11000 Belgrade, Serbia

**Keywords:** sarcopenia, muscle function, Crohn’s disease

## Abstract

Sarcopenia in inflammatory bowel disease can significantly influence disease course. Since current assessment focuses mainly on muscle quantity, we aimed to evaluate muscle function in Crohn’s disease (CD) patients and identify those at increased risk of muscle function impairment. This cross-sectional study included 84 patients with CD (76.2% male, mean age 35 ± 11 years) receiving infliximab. Body composition was assessed by bioelectrical impedance analysis, muscle strength by handgrip strength (HGS), and physical performance by gait speed. Half of the cohort demonstrated reduced muscle strength relative to age- and sex-adjusted norms, while none met criteria for confirmed sarcopenia. One third of the patients had low physical performance and only one patient screened positive for sarcopenia. In multivariable linear regression, sex was the strongest independent predictor of HGS (*p* < 0.001), followed by ileocolonic disease localization, which was independently associated with lower HGS compared to ileal (*p* = 0.045) and colonic (*p* = 0.046). Hemoglobin remained an independent predictor of HGS after multivariable adjustment (*p* = 0.021). Low muscle strength is common in patients with CD treated with infliximab, even in the absence of low muscle mass. Ileocolonic disease localization and lower hemoglobin levels are predictors of low muscle strength, highlighting the importance of early and comprehensive muscle function assessment in this population.

## 1. Introduction

Inflammatory bowel diseases (IBD), encompassing Crohn’s disease (CD) and ulcerative colitis (UC), are chronic, inflammatory, relapsing diseases characterized by significant changes in body composition at all stages [1,2]. These alterations are a result of multiple factors, most notably chronic inflammation, malabsorption, malnutrition, lack of physical activity, and corticosteroid use [3,4]. Important alterations that can significantly influence the disease course, prognosis and treatment outcomes include skeletal muscle mass and muscle function changes which are usually assessed when sarcopenia screening is performed. Sarcopenia plays an increasingly acknowledged role in the management of IBD since it affects both prognosis and outcomes [1]. Prior research varies widely in its findings on the prevalence of sarcopenia in patients with IBD, the reports ranging from 20% to 70% [1,5]. The literature indicates that skeletal muscle mass depletion in these patients correlates with disease severity, poorer clinical outcomes including therapy failure, an increased need for surgery and a higher risk of complications, as well as greater healthcare costs [6]. The current data on sarcopenia in IBD are scarce due to small sample sizes, retrospective study designs, and an inadequate inclusion of functional muscle assessment [2]. A comprehensive evaluation of sarcopenia, with emphasis on functional assessment, is crucial, particularly given that impaired function alone may adversely affect post-operative outcomes [1].

While the role of biologic therapy in improving body composition parameters (BCP) is documented in other chronic inflammatory diseases (e.g., rheumatoid arthritis), the data on patients with IBD are still lacking [7]. The available, albeit limited, data show that biologic therapy, especially anti-tumor necrosis factor α (anti-TNFα) drugs, can improve BCP. TNF-α, known in the literature as cachectin, is one of the most important proinflammatory cytokines that has a direct catabolic effect on muscle protein metabolism and is considered a key driver of sarcopenia [3]. Given the well-established role of TNF-α in the pathogenesis of sarcopenia, the beneficial effects of anti-TNFα therapy on BCP are both expected and well documented in various chronic inflammatory diseases. Anti-TNFα agents may mitigate sarcopenia by reducing systemic inflammation and inhibiting muscle protein catabolism. However, data on the impact of anti-TNFα therapy on sarcopenia parameters in patients with IBD, as one of the first and most widely used biologic therapies, still remain limited [4]. Findings from the few prospective studies assessing the impact of anti-TNFα therapy on sarcopenia in IBD, particularly in CD, indicate a beneficial effect of biologic treatment on muscle mass indices [8].

Sarcopenia, according to the available data, can also be present in patients achieving clinical remission. Reduced skeletal muscle volume has been reported in 60% of patients with CD in remission [9]. In both UC and CD, micronutrient deficiencies and reduced muscle strength are common even in remission and may not be identified through standard malnutrition screening methods [10].

One of the long-term goals in the treatment of patients with IBD, besides clinical remission and mucosal healing, is the improvement of physical performance and quality of life. Sarcopenia plays a significant role in this [11], highlighting the importance of a holistic approach in the management of these patients.

Given the importance of sarcopenia in IBD, there is a growing need to identify effective interventions for its prevention and treatment. However, early detection of sarcopenia in clinical practice based on basic clinical data is challenging, as BMI, a traditionally accepted parameter, is inadequate for its assessment. The evaluation of muscle parameters, particularly muscle function, should be an integral part of the assessment of patients with IBD in order to achieve better treatment outcomes, prevent complications, and improve quality of life [12].

The existing literature indicates that sarcopenia is more frequently documented in patients with CD, compared to UC [1,5]. This may be attributed to the frequent involvement of the upper gastrointestinal tract in CD, particularly the small intestine, which is the primary site of absorption of macronutrients, vitamins, and minerals. In contrast, the large intestine, which is predominantly affected in UC, plays a primary role in water and electrolyte absorption and microbial fermentation, with a lesser direct impact on nutritional status. Consequently, small bowel involvement in CD predisposes patients to greater nutritional depletion and subsequent impairment of muscle mass and function [3,13,14].

We aimed to assess muscle function parameters in patients with CD receiving infliximab therapy, with a primary focus on muscle strength and identification of its clinical predictors. Additionally, body composition parameters were analyzed in the same cohort.

## 2. Materials and Methods

### 2.1. Study Design and Settings

We conducted a cross-sectional study in daily hospital of the Clinic for Gastroenterohepatology, University Clinical Centre of Serbia, from May 2024 to October 2024. The study included all consecutive eligible patients during a six-month period. All participants gave written informed consent before participating in this study. The study was conducted in accordance with the 1964 Declaration of Helsinki and its later amendments and all applicable Serbian laws and guidelines. The study was approved by the Ethics Committee of the Faculty of Medicine, University of Belgrade and by the Ethics Committee of the University Clinical Centre of Serbia.

### 2.2. Selection of Participants

Consecutive outpatients with confirmed CD diagnosis treated with infliximab in the daily hospital of the Clinic for Gastroenterohepatology were evaluated for inclusion in the study during the study period. Diagnosis of CD was based on clinical, endoscopic and histological criteria.

The inclusion criteria were age from 18 to 70 years, provided signed informed consent, completed questionnaires, confirmed CD diagnosis and current infliximab therapy.

The exclusion criteria were age > 70 years, refusal to participate in the study, pregnancy, recent upper extremity trauma, presence of cardiac pacemaker, active infection and current treatment with other biologic therapies.

We reviewed patients’ socio-demographic and clinical data from their medical history. Phenotypic classification of CD was performed based on the Montreal classification. Clinical remission status was interpreted based on the Harvey–Bradshaw Index (HBI), with patients additionally dichotomized into remission and active disease categories. The Simple Endoscopic Score–Crohn disease (SES-CD) was used for endoscopic remission status estimation.

A potential source of bias in this study is the single tertiary center design, where patients with CD may represent a more severe disease phenotype compared to those treated in general hospitals. This limitation is acknowledged and discussed further in the Limitations section. To minimize measurement bias, all assessments were performed under standardized conditions using calibrated instruments in the same setting.

### 2.3. Sarcopenia Screening

Although the “Strength, assistance with walking, rising from a chair, climbing stairs, and falls questionnaire’’ (SARC-F) was originally designed to assess the risk of sarcopenia in the elderly population, it was administered to the participants of this study to evaluate potential indicators of muscle weakness and functional impairment [5].

### 2.4. Body Composition Parameters

Bioelectrical Impedance Analysis (InBody170) was done to estimate BCP: body weight, body mass index (BMI), skeletal muscle mass (SMM), body fat mass (BFM), percent body fat (PBF), and fat-free mass (FFM). Dividing SMM, FFM and BFM by height squared we calculated skeletal muscle mass index (SMI), fat-free mass index (FFMI) and fat mass index (FMI). Body height was measured by anthropometer Secca (Seca709, Hamburg, Germany) with an accuracy of 0.1 cm. Low SMI was defined as values < 8.6 kg/m^2^ for males and <6.2 kg/m^2^ for females, based on normative data derived from a healthy French adult population set at −2 standard deviations below the mean reference value [15]. These cut-offs were considered appropriate given the geographic and demographic proximity between the French and Serbian populations and are consistent with the EWGSOP2 recommendation to use regional normative references [5]. All measurements were done at the same time and under the same conditions. The World Health Organisation (WHO) standard categories for BMI were used: <18.5 kg/m^2^ (underweight), 18.5–24.9 kg/m^2^ (normal weight), 25–29.9 kg/m^2^ (overweight), ≥30 kg/m^2^ (obese) [16].

### 2.5. Muscle Strength

Muscle strength was assessed by handgrip strength (HGS), measured using a Saehan hydraulic hand dynamometer (Model SH5001, Saehan Corporation, Changwon, Korea). Bilateral measurements were done with three trials per hand allowing 30–60 s of rest between trials. The obtained data were compared with age- and sex-adjusted reference values for the general population as the EWGSOP2 cut-off values for muscle strength were developed in older populations and were considered less appropriate for the predominantly younger patients included in this study. This approach is consistent with the EWGSOP2 recommendation to use normative references from healthy young adults whenever possible [17,18]. Patients were divided into two groups based on the presence of low HGS. We analyzed if there was a difference in BCP, laboratory markers, presence of clinical or endoscopic remission, disease phenotype, presence of extraintestinal manifestations (EIMs) and previous operations, based on the presence of normal or low HGS.

Sarcopenia was defined as the presence of both low muscle mass and low muscle strength. Patients with low muscle strength were classified as those with probable sarcopenia.

### 2.6. Physical Performance

Physical performance was measured using the gait speed test, the usual walking 4 m speed test. According to EWSOP2, walking speed values of ≤0.8 m/s were taken as cut-off values for low physical performance [5].

### 2.7. Physical Activity Assessment

Participants completed a standardized short version of the International Physical Activity Questionnaire (IPAQ), a widely recognized tool for assessing physical activity levels. The data obtained from the questionnaire were analyzed using metabolic equivalent (MET) units, allowing for the categorization of participants into low, moderate, and high physical activity levels based on established IPAQ scoring guidelines (<600 MET-minutes per week; 600–3000 MET-minutes per week and >3000 MET-minutes per week) [19].

### 2.8. Statistical Analysis

A statistical analysis was performed with IBM SPSS software ver. 26. Continuous variables are presented as mean ± standard deviation (SD) or median (range), as appropriate, and were compared between groups using Student’s *t*-test or the Mann–Whitney U test depending on data distribution. The Kruskal–Wallis test was used for comparisons across three or more independent groups. Fisher’s exact test (Monte Carlo simulation) was used for categorical variables when expected cell counts were low.

Univariable linear regression analysis was performed to examine the associations between the clinical characteristics and muscle function and BCP as continuous dependent variables (HGS, SMI, PBF, FFMI, FMI and gait speed). Linear regression was chosen as the appropriate statistical method given the continuous nature of the dependent variables. Each clinical characteristic was entered as an independent variable in a separate model. For categorical independent variables with more than two levels (disease localization, behavior, age at diagnosis), dummy coding was applied. Results are presented as unstandardized regression coefficients (B), standardized coefficients (β), 95% confidence intervals (95% CI), and *p*-values. Only statistically significant associations of univariable linear regression are presented.

Multivariable linear regression analyses were subsequently performed for each continuous dependent variable. Models included clinical variables that were significant in univariable analysis as predictors, along with the following confounders: sex, age, BMI, disease duration, remission status and physical activity level. The multivariable linear regression was done since dichotomizing continuous variables into a binary outcome could reduce its variability and limit the analysis. Model fit was evaluated using R^2^ and adjusted R^2^ and the overall F-statistic with corresponding *p*-value. Results of the linear regression models are presented as unstandardized regression coefficients (B), standardized coefficients (β), 95% confidence intervals (CI), and *p*-values. A *p*-value of less than 0.05 was considered statistically significant for all analyses. Missing data were not imputed; complete case analysis was used. Data on FC were available for 77 of 84 patients, and SES-CD scores for 72 of 84 patients. All other variables were complete.

## 3. Results

### 3.1. Demographic and Clinical Data

This study included a total of 84 participants with CD (76.2% male), mean age 35 ± 11 years. All eligible patients meeting the inclusion criteria during the study period were enrolled. None of the eligible patients declined participation. Clinical and sociodemographic characteristics of patients stratified by HGS into low and normal HGS groups are presented in Table 1. Age differed significantly between low and normal HGS groups (*p* = 0.001). Additionally, the low HGS group had a significantly higher proportion of non-smokers than the normal HGS group (66.7% vs. 42.9%, *p* = 0.048). The proportion of patients diagnosed with CD after the age of 40 tended to differ between the low and normal HGS groups; however, this did not reach statistical significance (Fisher’s exact test, *p* = 0.057), and should be interpreted with caution given the small number of patients in these subgroups. No significant differences were observed between the groups in terms of physical activity levels, disease phenotype (localization, behavior, perianal disease), prior surgeries, EIMs, clinical and endoscopic remission status, disease and therapy duration.

### 3.2. Handgrip Strength According to Clinical Characteristics

Table 2 presents median HGS values stratified by all major clinical characteristics. Statistically significant differences were observed for sex (*p* < 0.001), disease localization (*p* = 0.002), smoking status (*p* = 0.021), and immunosuppressant-refractory status (*p* = 0.038). No significant differences were found for disease behavior, perianal disease, age at diagnosis, BMI category, physical activity level, clinical or endoscopic remission status, steroid dependence or refractoriness, EIMs, prior surgery, infliximab protocol, or gait speed category.

### 3.3. Assessment of Body Composition and Muscle Performance Parameters

Only one participant had a positive SARC-F screening result, indicating suspected sarcopenia. Half of the cohort exhibited reduced muscle strength relative to age- and sex-adjusted population norms (probable sarcopenia). However, none of the patients met the full criteria for sarcopenia, as no patient had low SMI. Additionally, one third of patients had low gait speed.

Table 3 presents body composition parameters, muscle performance measures, and results of the SARC-F questionnaire. Although most body composition measures did not differ significantly between the groups, BMI (*p* = 0.034) and SMI (*p* = 0.044) were significantly higher in patients with normal HGS. Gait speed did not differ significantly between groups. Six percent (*n* = 5) of patients were underweight, 44.0% (*n* = 37) had healthy weight, 36.9% (*n* = 31) were overweight, and 13.1% (*n* = 11) were obese.

### 3.4. Laboratory Findings 

The laboratory findings are shown in Table 4. We observed statistically significantly higher values of total cholesterol in the group of patients with normal HGS (*p* = 0.014), compared to those with low HGS, while other laboratory parameters such as hemoglobin (Hgb), serum iron, ferritin, C-reactive protein (CRP), albumin, triglycerides and fecal calprotectin (FC) did not differ significantly.

### 3.5. Associations Between Disease Characteristics and Body Composition and Muscle Function

Table 5 presents the results of an univariable linear regression analysis assessing associations between disease characteristics (including disease localization, age at diagnosis, treatment history, refractoriness to immunosuppressive therapy and laboratory parameters) and body composition and muscle function measures. Only variables showing statistically significant associations are displayed.

In univariable linear regression analysis, disease localization showed several significant associations. Ileocolonic CD was associated with lower HGS compared with both ileal (B = −6.439, 95% CI −11.802; −1.077, *p* = 0.019) and colonic disease (B = −10.062, 95% CI −15.711; −4.414, *p* = 0.001). SMI was higher in colonic than ileal CD (B = 1.219, 95% CI 0.045; 2.393, *p* = 0.042). However, the most pronounced association was observed between ileocolonic and colonic CD, where ileocolonic localization was strongly associated with lower SMI (B = −1.706, 95% CI −2.704; −0.707, *p* = 0.001). PBF showed a borderline association comparing colonic vs. ileal localization (B = −6.714, 95% CI −13.591; 0.163, *p* = 0.056) while proximal involvement was associated with higher PBF compared to colonic localization (B = 6.875, 95% CI 0.343; 13.407, *p* = 0.039). Proximal disease involvement was also associated with higher gait speed compared to ileal localization (B = 0.152, 95% CI 0.024; 0.279, *p* = 0.021).

Univariable linear regression also indicated associations between age at diagnosis and gait speed. Patients diagnosed at ages 16–40 (A2) had a lower gait speed compared to those diagnosed before 16 years of age (A1) (B = −0.150, 95% CI −0.266; −0.034, *p* = 0.012). However, those diagnosed after 40 years of age (A3) had a higher gait speed than the A2 group (B = 0.179, 95% CI 0.046; 0.313, *p* = 0.009).

Immunosuppressant-refractory status was negatively associated with HGS (B = −5018, 95% CI −9.020; −1.017, *p* = 0.015) and FFMI with borderline significance (B = −1.214, 95% CI −2.448; 0.019, *p* = 0.054).

Patients receiving infliximab as first-line biologic therapy had a higher FMI compared to those who were given infliximab as second-line biologic therapy (B = 2.618, 95% CI 0.461; 4.776, *p* = 0.018).

**Figure 1:**
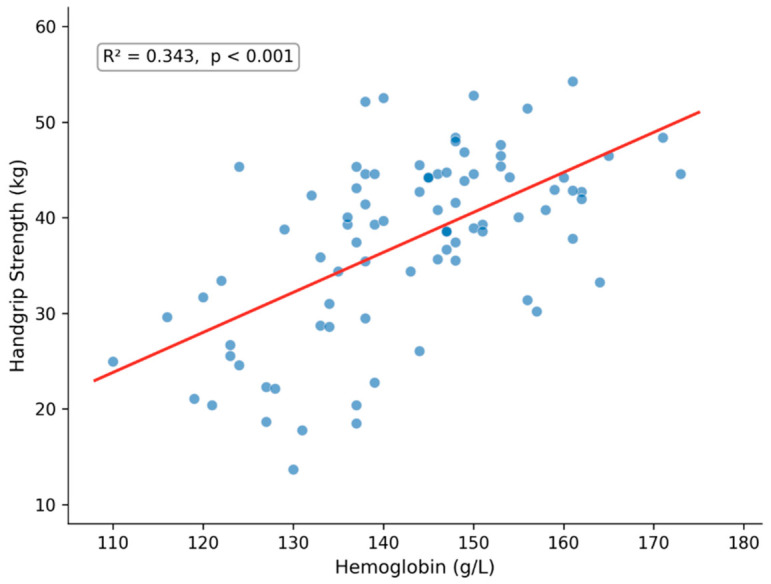
Scatter plot showing the association between hemoglobin (g/L) and handgrip strength (kg) in patients with Crohn’s disease on infliximab therapy (*n* = 84). The red line represents the linear regression fit. R^2^ = 0.343, *p* < 0.001.

Laboratory markers—Hgb and FC—showed strong associations with body composition parameters. Hemoglobin was positively associated with HGS (B = 0.418, 95% CI 0.291; 0.546, *p* = 0.000) as illustrated in Figure 1 and SMI (B = 0.061, 95% CI 0.037; 0.085, *p* = 0.000). Fecal calprotectin was inversely associated with FFMI (B = −0.001, 95% CI −0.002; 0.000, *p* = 0.034).

Multivariable linear regression was subsequently performed to identify independent predictors while adjusting for confounders.

**Figure 2:**
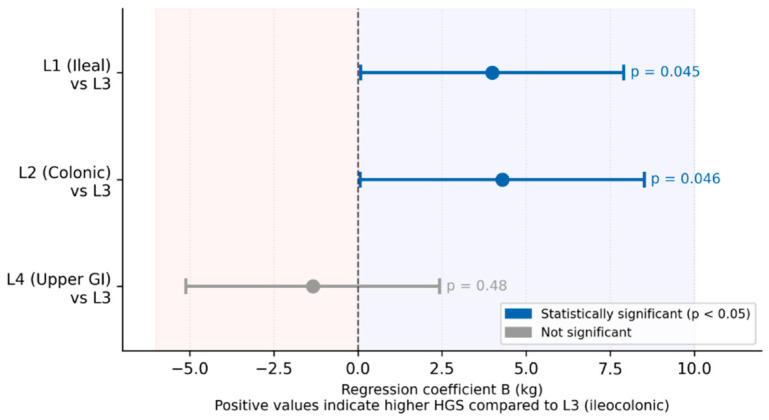
Multivariable linear regression coefficients (B) with 95% confidence intervals for disease localization as predictor of handgrip strength (HGS). Blue shading represents positive B coefficients, and red shading represents negative B coefficients. Reference category: L3 (ileocolonic localization). Positive values indicate higher HGS compared to L3. Model adjusted for sex, age, BMI, disease duration, remission status, physical activity, immunosuppressant-refractory status, and hemoglobin. HGS—handgrip strength; BMI—body mass index.

**Figure 3:**
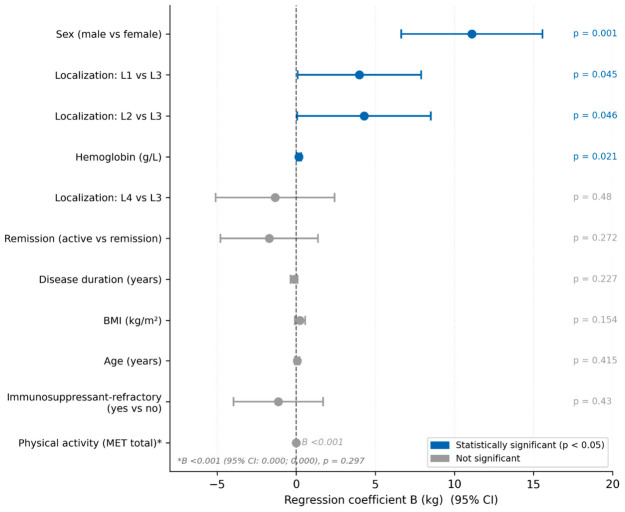
Forest plot of multivariable linear regression coefficients (B) with 95% confidence intervals for all predictors of handgrip strength. Blue markers indicate statistically significant predictors (*p* < 0.05). Reference categories: female sex, L3 (ileocolonic) localization, remission, no immunosuppressant-refractory status. Model: R^2^ = 0.639, F (11,72) = 11.561, *p* < 0.001. * Physical activity coefficient B < 0.001 (95% CI: 0.000; 0.000), *p* = 0.297.

The multivariable linear regression model significantly predicted HGS values (Table 6). The model explained 63.9% of variance (F (11,72) = 11.561; *p* < 0.001). Sex was the strongest independent predictor (B = −11.100; 95% CI −15.564; −6.636; *p* < 0.001), with male patients demonstrating significantly higher HGS values. Disease localization remained a significant predictor after adjustment confirming that this association is independent of confounders: patients with ileal (L1 vs. L3: B = 3.994; 95% CI 0.088; 7.900; *p* = 0.045) and colonic localization (L2 vs. L3: B = 4.288; 95% CI 0.069; 8.507; *p* = 0.046) had significantly higher HGS compared to patients with ileocolonic disease (Figure 2). Hemoglobin also remained an independent predictor but with a reduced effect size indicating partial confounding (B = 0.167; 95% CI 0.026; 0.309; *p* = 0.021). Immunosuppressant-refractory status lost statistical significance, suggesting the original association was confounded by other variables. Age, BMI, disease duration, physical activity level (MET total), and remission status were not significant predictors in the adjusted model. A forest plot summarizing the multivariable linear regression model with all predictors of HGS is shown in Figure 3.

**Figure 4:**
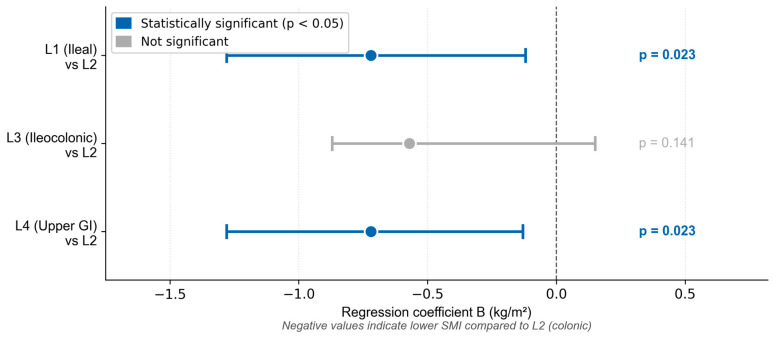
Multivariable linear regression coefficients (B) with 95% confidence intervals for disease localization as predictor of skeletal muscle mass index (SMI). Reference category: L2 (colonic localization). Negative values indicate lower SMI compared to L2. Model adjusted for sex, age, BMI, disease duration, remission status, physical activity, and hemoglobin. SMI—skeletal muscle mass index; BMI—body mass index.

Multivariable linear regression analysis for SMI is shown in Table 7. The model significantly predicted SMI values (F (10,73) = 27.553; *p* < 0.001), explaining 79.1% of variance. Sex (B = −1.957; 95% CI −2.538; −1.376; *p* < 0.001) and BMI (B = 0.226; 95% CI 0.183; 0.270; *p* < 0.001) were the strongest independent predictors. Disease localization also remained significant: ileal (L1 vs. L2: B = −0.722; 955CI −1.340; −0.105, *p* = 0.023) and proximal localization (L4 vs. L2: B = −0.708; 95% CI −1.314; −0.102, *p* = 0.023) were associated with lower SMI compared to colonic disease. Proximal localization became significant only after adjustment, representing a new finding not present in the univariable analysis while L3 vs. L2 lost significance indicating partial confounding by sex and BMI (Figure 4).

The multivariable linear model for gait speed showed borderline overall significance (F (11,72) = 1.729; *p* = 0.084), explaining 20.9% of variance (Table 8). Proximal disease involvement (L4 vs. L1: B = 0.137; 95% CI 0.007; 0.267; *p* = 0.040) and older age at diagnosis (A3 vs. A2: B = 0.204; 95% CI 0.022; 0.386; *p* = 0.029) were significant independent predictors of higher gait speed. Indicating slower walking speed in patients with ileal disease and those diagnosed between 16 and 40 years of age. The difference between A1 (diagnosed before 16) and A2 lost statistical significance after adjustment (*p* = 0.067).

Table 9 presents the results of the multivariable linear regression analyses for the remaining body composition parameters (PBF, FFMI, and FMI). All three models reached overall statistical significance (*p* < 0.001), explaining 75.0%, 67.8%, and 81.2% of variance, respectively. Regarding FFMI, Sex (Beta = −0.490; 95% CI −4.030; −2.162; *p* < 0.001) and BMI (Beta = 0.509; 95% CI 0.229; 0.413; *p* < 0.001) were the strongest predictors, with female patients having lower FFMI, and higher BMI associated with higher FFMI. Disease duration emerged as a significant independent predictor in the multivariable model (B = −0.085; 95% CI −0.146; −0.025; *p* = 0.007), with longer disease duration associated with lower FFMI. Fecal calprotectin remained a significant predictor after adjustment (B = −0.001; 95% CI −0.002; 0.000; *p* = 0.024). Immunosuppressant-refractory status lost significance after adjustment, while remission, physical activity, and age were not significant.

**Figure 5:**
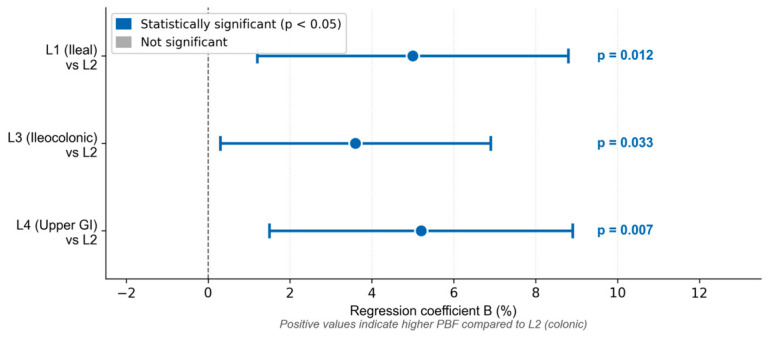
Multivariable linear regression coefficients (B) with 95% confidence intervals for disease localization as predictor of percent body fat (PBF). Reference category: L2 (colonic localization). Positive values indicate higher PBF compared to L2. Model adjusted for sex, age, BMI, disease duration, remission status, and physical activity. PBF—percent body fat; BMI—body mass index.

For PBF, Sex (B = 12.555; 95% CI 9.916; 15.195; *p* < 0.001) and BMI (B = 1.382; 95% CI 1.118; 1.645; *p* < 0.001) were also the strongest independent predictors. Disease localization remained significant after adjustment, with ileal (L1 vs. L2: *p* = 0.012), ileocolonic (L3 vs. L2: *p* = 0.033), and proximal localization (L4 vs. L2: *p* = 0.007) all associated with higher PBF compared to colonic disease (Figure 5). For another adiposity parameter, FMI, BMI was the dominant predictor (B = 0.631; 95% CI 0.553; 0.710; *p* < 0.001), followed by sex (B = 3.154; 95% CI 2.375; 3.934; *p* < 0.001), and disease duration (B = 0.059; 95% CI 0.005 to 0.114; Beta = 0.136; *p* = 0.034). Line of therapy (2nd vs. 1st line infliximab) lost statistical significance after adjustment (*p* = 0.260 vs. *p* = 0.018 in univariable analysis), indicating this association was confounded by sex, BMI, and disease duration. Disease duration emerged as a significant independent predictor only in the multivariable model.

## 4. Discussion

### 4.1. Muscle Function and Body Composition Findings

Sarcopenia is a key determinant of clinical outcomes in CD, influencing disease progression, treatment response, and overall prognosis. A recent meta-analysis indicated that 52% of patients with CD and 37% with UC have sarcopenia [1]. The higher incidence could be attributed to the involvement of the upper gastrointestinal tract, particularly the small bowel, which represents the primary site of macronutrient, vitamin, and mineral absorption [3,13,20]. Additionally, the chronic nature of CD contributes to disease-related complications including stricturing disease, fistula formation and surgical resections resulting in short bowel syndrome. The heterogeneity observed in previously published results is primarily explained by different methods used for assessing sarcopenia, mainly due to the lack of a standardized algorithm. Additionally, these results are influenced by the diversity of the studied populations (European, Asian), disease phenotype and remission status, as well as different therapeutic approaches [1,2,3].

While most previous studies focused predominantly on muscle mass when describing sarcopenia and reporting its prevalence [3], more recent research tries to address both anatomical and functional muscle components. A recent systematic review reported that the prevalence of probable sarcopenia (low muscle strength) was approximately 34% in patients with IBD (35% in CD and 32% in UC), while the prevalence of sarcopenia (defined as combined low muscle mass and strength) was lower and reported as 17% [21]. In our study, none of the patients had low SMI based on European population reference values, and consequently none met the criteria for sarcopenia. This finding should be interpreted in the context of our cohort, which consisted of a male-predominant group of patients with CD treated with infliximab at a tertiary center. The majority of patients were in remission, with a median infliximab therapy duration of 3.1 years (range 0.1–8.1), which may also reflect a relatively well-controlled disease state. All of these factors represent characteristics that may have influenced muscle mass parameters. On the other hand, 50% of our cohort had low HGS in reference to a healthy European population.

Our findings are also consistent with those reported by Ergenc I et al. [18] in an outpatient cohort of 129 patients with IBD (63 CD and 66 UC) evaluated at a tertiary center, where disease activity was present in 27% of patients with CD and 29% of those with UC. Using EWGSOP2 criteria with ethnicity-based population thresholds, the authors reported that 34.9% of patients with IBD had probable sarcopenia (defined as low muscle strength), while the prevalence of confirmed sarcopenia remained low (approximately 2%). These findings align with our own, reinforcing the concept that, in younger adult IBD populations, reduced muscle strength is considerably more prevalent than overt loss of muscle mass, and that probable sarcopenia may represent the predominant functional phenotype in this setting. Forty percent of these patients were treated with biologics. This supports the value of objective strength assessment as a more sensitive indicator of early functional impairment in this population.

Similarly, in one of the few prospective studies, Dermine et al. [22] evaluated 60 outpatients with IBD, predominantly treated with biologic therapy (97%) and with longer disease durations, and reported a prevalence of probable sarcopenia and sarcopenia of 18% and 10%, respectively. Our findings are in line with these results, as the prevalence of probable sarcopenia remains higher than that of confirmed sarcopenia.

Muscle function is a critical prognostic factor in these patients, as it is shown that it declines more rapidly than muscle mass and may serve as an early indicator for the assessment and prevention of sarcopenia [23]. Moreover, muscle strength is now considered a better predictor of adverse outcomes [24]. Reduced muscle function has been reported in patients with CD even during clinical remission. Van Langenberg et al. evaluated 41 patients with CD in clinical remission and found significantly lower strength performance compared to age-matched healthy controls, despite no significant differences in weight, BMI, fat mass, or fat-free mass. This further supports our observation that reduced muscle strength may occur independently of preserved muscle mass and aligns with our finding that 50% of our cohort exhibited low HGS, despite the fact that majority of patients were in disease remission [25].

The SARC-F questionnaire was originally designed and validated in elderly populations, where it demonstrated adequate correlation with grip strength and predictive validity for adverse functional outcomes [26]. In our cohort, with a mean age of 35 years, only one patient screened positive on SARC-F, while 50% had reduced handgrip strength. This clinically significant discordance highlights the limited sensitivity of SARC-F in younger populations with CD and suggests that objective measure of muscle strength, such as handgrip dynamometry, may be more appropriate screening tool in this group of patients. This is also consistent with recent evidence demonstrating poor diagnostic accuracy of SARC-F in populations with IBD [18,27]. Furthermore, even in older adult populations for which SARC-F was originally developed, the conventional cut-off of ≥4 has demonstrated insufficient sensitivity, with lower thresholds proposed to improve early detection [28].

Another finding of our study was that one third of patients had low gait speed, despite a relatively young mean age and preserved muscle mass which further supports the presence of functional impairment in this cohort. Gait speed is a powerful predictor of adverse outcomes including falls, hospitalization, and mortality in elderly populations [29], but its prognostic significance in younger cohorts with IBD remains unclear. According to the EWGSOP2 guidelines, physical performance measures such as gait speed are used to assess the severity of established sarcopenia [30]. However, physical performance is a multidimensional concept that reflects whole-body function related to movement, encompassing not only muscle function but also the function of the central and peripheral nervous system and balance [31]. Our findings suggests that impaired physical performance may occur in patients with CD receiving biologic therapy independently of sarcopenia, even in the setting of well-controlled disease and preserved muscle mass, and warrants further investigation.

### 4.2. Clinical Predictors of Muscle Function and Body Composition

Existing evidence indicates that HGS is associated with age showing a decline with advancing years [32]. In our cohort, however, the mean age was significantly higher in the normal HGS group (*p* = 0.001), whereas patients classified as having low HGS were younger than those with normal HGS. This finding should be interpreted while bearing in mind our HGS classification—handgrip strength categories in our study were defined using age-specific reference values. Therefore, the finding that the normal HGS group was older indicates that older patients more often had grip strength within the expected range for their age, whereas younger patients more often had grip strength below the expected range for age-matched healthy individuals. This finding may point to early functional impairment in complicated CD phenotypes that are usually treated with infliximab, potentially related to cumulative disease burden, inflammation-related catabolism, nutritional deficits, or treatment effects. The results should be interpreted with caution, taking into account both the age-specific definition of HGS categories and the difference in age distribution between groups.

Cigarette smoking is a well-recognized risk factor in patients with CD and has been associated with a more aggressive disease course leading to increased therapeutic needs and higher relapse rates in patients who continue smoking after diagnosis [33]. Beyond its role in CD, smoking has also been proposed as a potential contributor to sarcopenia in the general population. A recent systematic review and meta-analysis reported an association between smoking and sarcopenia. However, the overall certainty of evidence was judged as very low due to the observational nature of available studies, substantial heterogeneity, and inconsistent definitions of smoking exposure [34]. Interestingly, in our cohort, the low HGS group had a significantly higher proportion of non-smokers compared with the normal HGS group. Also, current smokers had a significantly higher median HGS compared to non-smokers. This result should not be interpreted as a protective effect of smoking on muscle strength. A more likely explanation is that the distribution of smoking status is not independent of sex in this cohort. Given the male predominance and the substantially higher HGS in male patients, subgroups with a higher proportion of male patients will naturally demonstrate higher raw HGS values regardless of the clinical characteristic under examination. Finally, because the statistical significance was borderline (*p* = 0.048), this finding warrants cautious interpretation and should ideally be confirmed in larger samples with age-adjusted analyses.

Previous studies suggest that HGS is more useful than BMI for identifying low lean mass and reduced muscle mass, since BMI is strongly influenced by fat mass and can therefore mask muscle mass loss [30,35]. In our cohort, SMI was higher in the normal HGS group (*p* = 0.044), which is consistent with the close link between muscle mass and muscle function [36]. BMI was also higher in the normal HGS group (*p* = 0.034).

In addition, in our multivariable analyses, BMI emerged as a significant independent predictor of higher SMI, FFMI, PBF, and FMI, confirming its role as a comprehensive marker reflecting both lean and fat body compartments [5]. This is in line with previous reports indicating that BMI is associated with multiple body composition parameters in IBD population [37].

However, BMI should be interpreted as a non-specific, crude indicator of body composition [5] since BMI-based anthropometric assessment in IBD patients may misrepresent body composition, meaning that an excess of fat mass can mask lean mass deficits.

This consideration is particularly relevant in our cohort, as anti-TNFα therapy has been shown to improve BCP in patients with CD [38,39] which may result in apparent BMI normalization while subtle functional impairments in muscle strength persist. In their study on correlation of BCP and long-term outcomes in patients with CD after anti-TNFα treatment, Ando K. et al. reported that an early application of anti-TNFα therapy significantly affected skeletal muscle mass, fat mass and bone mineral content, supporting patients’ long-term nutritional status and reducing the probability of malnutrition [38]. This supports the use of more specific measures such as bioelectrical impedance analysis and handgrip strength alongside BMI, particularly in patients with CD receiving biologic therapy, where altered body composition may not be adequately reflected in BMI alone.

Among laboratory parameters, total cholesterol was the only variable that differed significantly between groups, with higher values in the normal HGS group (4.62 ± 1.07 vs. 4.09 ± 0.86 mmol/L; *p* = 0.014), although mean values in both groups were within the laboratory reference range (<5.2 mmol/L). Lower cholesterol can be seen in the context of chronic inflammation or undernutrition, which may coexist with reduced muscle strength in patients with CD [40]. Since CRP, albumin, and fecal calprotectin were comparable between groups, this difference is unlikely to reflect higher inflammatory activity. It may instead relate to differences in nutritional status between groups, as the normal HGS group also had higher BMI and SMI. Although statistically significant, this difference is likely of marginal clinical significance.

Handgrip strength was significantly associated with several clinical and biological factors.

In our male-predominant cohort (76.2% male), male sex emerged as the strongest independent predictor of higher HGS in the multivariable model, consistent with well-established sex differences in muscle strength observed across populations [41]. Observed difference in absolute HGS values between sexes, underscores the necessity of using sex-specific reference values when classifying patients as having low or normal HGS. Notably, despite male predominance typically associated with higher muscle strength, half of our cohort still exhibited reduced HGS, highlighting the burden of muscle function impairment in patients with CD on biologic therapy. Similarly, male sex was an independent predictor of higher SMI and FFMI, while female sex was associated with higher PBF and FMI in our multivariable analyses, supporting previous evidence on sex-based differences in body composition [42].

Secondly, disease localization showed a clinically relevant association with HGS after adjustment for age, sex, BMI, disease duration, remission status, and physical activity level. Ileocolonic involvement was associated with lower HGS values compared to isolated ileal and colonic disease. This finding indicates that ileocolonic CD represents the phenotype most vulnerable to impaired muscle strength, independent of other confounders. The more extensive inflammatory burden of ileocolonic disease, combined with the compromised function of both the ileum as the primary site of nutrient absorption [13] and the colon, which contributes to metabolic homeostasis through short-chain fatty acid production, water and electrolyte absorption [20], may collectively drive this muscle strength impairment. In addition, the ileocolonic form of CD often necessitates more aggressive medical treatment, including corticosteroids, which are known to accelerate muscle breakdown [14,43].

The association of ileocolonic disease with lower HGS values appears to be contradicted by the raw medians where patients with ileocolonic localization had the highest raw median HGS and colonic localization the lowest. However, this discrepancy is a direct consequence of the uneven sex distribution across disease localization subgroups. In our cohort, ileocolonic disease is the most common phenotype (*n* = 37), and given the 76.2% overall male prevalence, this group is likely to contain the highest absolute number of male patients, whose substantially higher HGS inflates the raw median for L3. After adjustment for sex, BMI, and other confounders in the multivariable model, the relationship reverses.

Regarding disease localization, different patterns of involvement emerged as independent predictors of BCP in our multivariable analyses.

An interesting and clinically relevant observation emerged when comparing predictors of muscle strength and muscle mass across different CD localizations. In multivariable analysis ileal and proximal disease were independently associated with relatively lower SMI values compared to colonic localization. This is consistent with the absorptive role of the small bowel and with previous reports showing that small bowel involvement predisposes patients with CD to greater nutritional depletion and subsequent muscle mass reduction [2,44]. Since no patient in our cohort met the criteria for low SMI these findings should be interpreted as subtle shifts in body composition within the normal range rather than clinically overt muscle mass deficits. In that sense, small bowel involvement appeared to primarily affect muscle mass, whereas combined small and large bowel involvement had the greatest impact on muscle strength, relative to isolated ileal or colonic disease. This apparent dissociation between muscle mass and muscle strength across disease phenotypes supports the concept of dynapenia, in which muscle quality and functional capacity may be compromised independently of quantitative muscle mass [29,45]. These findings may reflect distinct mechanisms underlying muscle impairment across CD localizations where ileocolonic involvement may primarily affect muscle quality and function through systemic inflammation [46] while small bowel involvement may primarily affect muscle mass through impaired nutrient absorption [13]. This underscores the value of assessing both muscle mass and function in CD, as neither alone fully captures the spectrum of muscle impairment.

For gait speed, proximal disease involvement was associated with higher walking speed compared to ileal localization, although this finding should be interpreted with caution given the heterogeneity within the L4 category and the relatively small subgroup sizes.

In a recent systematic review Ding NS et al. [47] identified only two studies that examined the relationship between CD localization and BCP [44,48]. They reported that ileal and ileo-colonic disease were associated with decreased fat mass compared to other distributions. Our results are in contrast with these, suggesting ileal, ileocolonic, and proximal disease to be associated with higher PBF compared to colonic localization. Our cohort consisted of patients with CD on established infliximab therapy, which has been shown to improve nutritional status and body composition, potentially modifying fat distribution over time [49]. Therefore, our study is one of the few contemporary investigations that associated disease localization with BCP.

Hemoglobin was also an independent predictor of HGS in the adjusted model supporting the concept that anemia, frequently observed in patients with CD due to chronic inflammation, iron deficiency, or malabsorption, may contribute to reduced oxygen delivery and impaired muscle function [6,50].

Disease duration emerged as a significant independent predictor in the multivariable analyses, with an opposing effect on lean and fat compartments. In line with previous findings, longer disease duration was associated with lower FFMI, but higher FMI [51]. This pattern suggests a progressive shift in body composition over the course of treated CD, characterized by gradual loss of lean mass and concomitant accumulation of fat mass. Such changes may reflect the cumulative impact of chronic inflammation, nutritional depletion, reduced physical activity, and the long-term effects of corticosteroid exposure as well as effects of anti-TNFα therapy on body composition [5,39,40]. Schneider et al. observed, negative correlation between disease duration and fat-free body mass in patients with CD in remission [9,36].

In line with this, another predictor of lower FFMI values was FC as a marker of active intestinal inflammation, supporting the role of ongoing chronic inflammatory activity in driving lean tissue depletion [46]. Together, these findings indicate that both cumulative disease exposure (disease duration) and current inflammatory burden expressed through FC may be related to reduced FFMI values in our cohort. Importantly, disease duration was not an independent predictor of SMI, which may indicate that these gradual changes first become detectable in the broader FFMI measure, reflecting overall lean tissue including both skeletal and smooth muscle, organs, and body water. Also, in our cohort of patients with CD on infliximab therapy, disease control and biologic treatment may partially mitigate the loss of skeletal muscle mass while more subtle changes in overall lean tissue composition persist. This finding underscores the importance of longitudinal body composition monitoring in CD patients, particularly those with long-standing disease, even when overall muscle mass appears preserved.

Although previously reported findings suggested that both early and late disease onset may predispose patients to reduced muscle performance through distinct biological and behavioral pathways [52,53,54], our findings were not fully in accordance with them. In our multivariable analyses patients diagnosed with CD between 16 and 40 years of age had significantly lower gait speed compared to those diagnosed after the age of 40, an association that remained significant in linear regression model, after adjustment for confounders. One possible explanation is that this age group experiences the longest cumulative exposure to active disease during a physiologically demanding period of life, when occupational, academic, and social responsibilities may limit opportunities for recovery and physical activity [55]. Older adults may present with more stable disease course or lower functional expectations [56]. However, these findings should be interpreted with caution. The overall linear regression model for gait speed did not reach statistical significance (*p* = 0.084), but this finding needs confirmation in larger cohorts.

Early use of anti-TNFα therapy is known to play a pivotal role in improving the long-term outcomes of Crohn’s disease. Our results show that patients who were started on biologic therapy in a step-up approach due to the immunosuppressive-refractory disease have lower FFMI and HGS, compared to those who were treated with infliximab as part of a top-down approach. However, these results, implying the benefits of early anti-TNFα therapy initiation on muscle mass and function parameters, did not retain statistical significance in multivariate linear models. Additional research involving larger cohorts is warranted to clarify and confirm these observations.

Our findings did not support a significant association between clinical remission status and muscle function or BCP, as remission did not emerge as an independent predictor in our multivariable analyses. This contrasts with previous reports linking disease activity to impaired body composition and muscle function in IBD patients [36,57]. However, this discrepancy may be explained by the predominance of patients in clinical remission within our cohort, which limited the variability of this variable and may have reduced the ability to detect significant associations.

### 4.3. Limitations

This study has several limitations that should be considered when interpreting the findings. The study population consisted exclusively of patients receiving infliximab therapy with a male predominance, which limits the generalizability of our findings to the broader population of patients with CD. Additionally, the cross-sectional design precludes causal conclusions regarding the observed associations. Second, several subgroup analyses in this study were based on small sample sizes. These analyses may have been underpowered to detect true differences, increasing the risk of Type II errors. Conclusions drawn from these subgroups should therefore be interpreted with caution, and the findings should be confirmed in larger prospective cohorts. Third, the normative reference values used for handgrip strength classification were derived from British population cohorts and those used for skeletal muscle mass index from a French population cohort. Although all populations are of European origin, the applicability of these reference values to a Serbian population has not been formally validated, which represents a potential source of misclassification. A further limitation of this study is the absence of nutritional assessment, including dietary intake data and validated nutritional screening tools which limits a more comprehensive evaluation of the role of malnutrition in the observed associations. Finally, although BIA is a widely used and accessible method for body composition assessment, it has some limitations in patients with IBD due to potential fluid shifts and altered hydration status, which may affect the accuracy of measurements.

## 5. Conclusions

Sarcopenia and muscle dysfunction in patients with CD are gaining recognition as a key area of research, emphasizing the need for early detection and targeted interventions to mitigate adverse clinical outcomes.

Our study demonstrated that the presence of low muscle strength is common in patients with CD treated with infliximab even in the absence of low muscle mass or sarcopenia. Female sex, ileocolonic disease localization compared to ileal and colonic, and lower hemoglobin levels were identified as independent predictors of low muscle strength. In addition, disease localization emerged as a significant independent predictor of body composition parameters, including lean and fat mass, with distinct patterns observed across different phenotypes. These subgroups may warrant closer monitoring and earlier, more proactive assessment of muscle function and body composition. Objective measures such as handgrip dynamometry and bioelectrical impedance analysis may complement traditional anthropometric parameters and facilitate early identification of muscle impairment.

These findings should be interpreted in the context of our selected tertiary-care cohort of male-predominant patients with CD on biologic therapy, and warrant confirmation in larger prospective studies.

## Figures and Tables

**Table 1:** Patients’ Characteristics and Clinical Background.

Characteristic	Total (*n* = 84)	Low HGS	Normal HGS	*p* Value
**Gender**				
Male	64	32	32	/
Female	20	10	10	/
**Age (years)**	35 ± 11	31 ± 10	39 ± 11	**0.001**
**Smoking status**				
Smoker	30	11	19	NS
Non-smoker	46	28	18	**0.048**
Ex-smoker	8	3	5	NS
**Age at diagnosis**				
A1 (<16 years)	11	8	3	NS
A2 (17–40 years)	65	33	32	NS
A3 (>40 years)	8	1	7	**0.057**
**Disease localization**				
L1	15	11	4	NS
L2	13	7	6	NS
L3	37	16	21	NS
L4	19	8	11	NS
**Disease behavior**				
B1	31	17	14	NS
B2	17	8	9	NS
B3	36	17	19	NS
**Perianal disease**				
Present	30	13	17	NS
Absent	54	29	25	NS
**Clinical activity (HBI)**				
Remission	57	26	31	NS
Mild	19	12	7	NS
Moderate	6	3	3	NS
Severe	2	1	1	NS
**Clinical remission**				
Yes	57	26	34	NS
No (mild, moderate, severe activity)	27	16	11	NS
**Steroid-refractory disease**				
Yes	5	2	3	NS
No	79	40	39	NS
**Steroid-dependent disease**				
Yes	13	6	7	NS
No	71	36	35	NS
**Immunosuppressant-refractory**				
Yes	36	17	19	NS
No	48	25	23	NS
**Extraintestinal manifestations**				
Present	22	13	9	NS
Absent	62	29	33	NS
* Musculoskeletal EIMs	16	10	6	NS
**Surgical history (operations)**				
Yes	31	14	17	NS
No	53	28	25	NS
**Protocol of IFX administration**				
Standardized	5	2	3	NS
Optimized	79	40	39	NS
**Line of biologic therapy**				
IFX—First-line biologic drug	74	37	37	NS
IFX—Second-line biologic drug	10	5	5	NS
* Previous biologic—ADA	9	5	4	NS
* Previous biologic—VDZ	1	/	1	NS
**Disease duration (years)**	7 (0.1–40)	6.5 (0.1–24)	7 (0.1–40)	NS
**IFX therapy duration (years)**	3.1 (0.1–8.1)	2.2 (0.1–7.3)	3.4 (0.1–8.1)	NS
**SES-CD**	3.8 (0–30)	1 (0–14)	3 (0–30)	NS
**Physical activity (MET categories)**				
Light	4	3	1	NS
Moderate	32	13	19	NS
Vigorous	48	26	22	NS

HBI—Harvey–Bradshaw index; IFX—infliximab; ADA—Adalimumab; VDZ—Vedolizumab; SES-CD—Simple Endoscopic Score for Crohn’s Disease; MET—Metabolic Equivalent of Task, HGS—hand grip strength; NS—non significant. * Data are presented as mean ± standard deviation or median (min–max). Bold *p*-values indicate statistical significance (*p* < 0.05).

**Table 2:** HGS values (kg) according to clinical characteristics.

Variable	*n*	Median (Min–Max)	*p*-Value
**Overall HGS (kg)**	84	39.31 (13.68–54.28)	—
**Sex**			**<0.001**
Male	64	42.15 (20.41–54.28)	
Female	20	24.76 (13.68–47.63)	
**Disease localization**			**0.002**
L1—Ileal	15	37.80 (18.67–51.41)	
L2—Colonic	13	30.99 (18.51–42.94)	
L3—Ileocolonic	37	42.71 (22.75–52.54)	
L4—Upper GI	19	40.07 (13.68–54.28)	
**Smoking status**			**0.021**
Smoker	30	42.79 (17.76–52.14)	
Non-smoker	46	35.60 (13.68–54.28)	
Ex-smoker	8	40.44 (20.41–52.54)	
**Immunosuppressant-refractory**			**0.038**
Yes	36	42.15 (17.76–54.28)	
No	48	37.61 (13.68–51.41)	
**BMI category**			0.074
Underweight	5	38.56 (18.67–44.23)	
Healthy weight	37	37.42 (13.68–45.51)	
Overweight	31	41.43 (20.40–54.28)	
Obese	11	39.31 (22.75–48.38)	
**Disease behavior**			0.586
B1—Inflammatory	31	39.31 (17.76–51.41)	
B2—Stricturing	17	40.82 (20.40–54.28)	
B3—Penetrating	36	39.11 (13.68–52.54)	
**Perianal disease**			0.493
Present	30	40.07 (13.68–51.41)	
Absent	54	39.11 (17.76–54.28)	
**Age at diagnosis**			0.173
A1 (<16 years)	11	38.92 (13.68–44.59)	
A2 (17–40 years)	65	39.31 (17.76–54.28)	
A3 (>40 years)	8	44.60 (20.41–52.54)	
**Physical activity (MET)**			0.311
Low (<600)	4	33.63 (20.41–42.33)	
Moderate (600–3000)	32	40.75 (13.68–52.77)	
High (>3000)	48	39.11 (18.67–54.28)	
**Clinical remission**			0.239
Yes	57	40.07 (13.68–54.28)	
No	27	38.56 (17.76–46.49)	
**Endoscopic remission**			0.931
Yes	51	39.31 (13.68–54.28)	
No	33	40.07 (18.51–48.38)	
**Steroid-dependent disease**			0.276
Yes	13	37.42 (18.51–51.41)	
No	71	40.07 (13.68–54.28)	
**Steroid-refractory disease**			0.395
Yes	5	44.23 (24.95–45.36)	
No	79	39.31 (13.68–54.28)	
**Extraintestinal manifestations**			0.295
Present	22	38.67 (17.76–46.49)	
Absent	62	40.44 (13.68–54.28)	
**Prior surgery**			0.732
Yes	31	39.31 (13.68–54.28)	
No	53	39.31 (17.76–52.14)	
**Protocol of IFX administration**			0.205
Standardized	5	44.60 (38.56–46.49)	
Optimized	79	39.31 (13.68–54.28)	
**Gait speed**			0.500
Low (<0.8 m/s)	28	38.18 (13.68–52.54)	
Normal (≥0.8 m/s)	56	39.88 (18.51–54.28)	

HGS—Handgrip strength; BMI—Body mass index; MET—Metabolic equivalent; IFX—infliximab. Data are presented as median (min–max). Bold *p*-values indicate statistical significance (*p* < 0.05).

**Table 3:** Body composition parameters and muscle performance parameters.

Body Composition Parameters	Total	Low HGS	Normal HGS	*p* Value
BMI kg/m^2^	25.11 ± 4.43	24.08 ± 4.02	26.13 ± 4.64	**0.034**
SMI kg/m^2^	10.66 ± 1.64	10.26 ± 1.44	11.18 ± 1.74	**0.044**
FFMI kg/m^2^	18.71 ± 2.85	18.1 ± 2.25	19.25 ± 3.30	0.086
FMI kg/m^2^	6.33 ± 3.31	5.88 ± 3.09	6.79 ± 3.48	0.207
PBF %	23.82 ± 9.24	23.41 ± 9.74	24.23 ± 8.81	0.687
**BMI categories**				0.276
Underweight	5	4	1	
Healthy weight	37	21	16	
Overweight	31	13	18	
Obese	11	4	7	
**Muscle performance**				
Gait speed (m/s)	0.88 ± 0.18	0.88 ± 0.21	0.87 ± 0.15	0.726
**SARC-F Questionnaire**				1.000
Sarcopenia suspected	1	0	1	
Sarcopenia not suspected	83	42	41	

BMI—body mass index; SMI—skeletal muscle mass index; FFMI—fat free mass index; FMI—fat mass index; PBF—percent body fat; SARC-F—strength, assistance, rise, climb, fall; HGS—hand grip strength. Data are presented as mean ± standard deviation or median (min–max). Bold *p*-values indicate statistical significance (*p* < 0.05).

**Table 4:** Laboratory findings in patients with low vs. normal HGS.

Laboratory Findings	Total	Low HGS	Normal HGS	*p* Value
Hgb g/L	142.78 ± 13.15	142.85 ± 12.56	142.71 ± 13.87	0.961
Fe µmol/L	13.2 (2–38.10)	14.65 (5–30.8)	11.95 (2–37.8)	0.731
Ferritin µg/L	47.65 (6.9–791.1)	30 (6.9–354.3)	58.4 (7.1–791.7)	0.354
CRP mg/L	1.3 (0.13–31.5)	1.45 (0.50–31.5)	1.15 (0.13–19.50)	0.322
Albumin g/L	45 (29–52)	45 (29–52)	45 (33–51)	0.989
Cholesterol mmol/L	4.36 ± 1.01	4.09 ± 0.86	4.62 ± 1.07	**0.014**
Tg mmol/L	1.14 (0.24–4)	1.05 (0.24–3.49)	1.15 (0.31–4)	0.217
FC µg/g	45 (0–2000)	45 (0–2000)	44.10 (0–2000)	0.771

Hgb—Hemoglobin; Fe—iron; CRP—C reactive protein; Tg—triglycerides; FC—fecal calprotectin; HGS—hand grip strength. Data are presented as mean ± standard deviation or median (min–max). Bold *p*-values indicate statistical significance (*p* < 0.05).

**Table 5:** Associations between disease characteristics and body composition and muscle function—results of univariable linear regression analysis.

Dependent Variable	Independent Variable	B	95% CI	Beta	*p*	R^2^	VIF
	**Localization**						
HGS (kg)	L3 vs. L1	−6.439	−11.802; −1.077	−0.342	0.019	0.156	1.9
	L3 vs. L2	−10.062	−15.711; −4.414	−0.534	0.001	0.156	2.1
SMI (kg/m^2^)	L2 vs. L1	1.219	0.045; 2.393	0.269	0.042	0.138	1.5
	L3 vs. L2	−1.706	−2.704; −0.707	−0.517	0.001	0.138	2.1
PBF (%)	L2 vs. L1	−6.714	−13.591; 0.163	−0.264	0.056	0.062	1.5
	L4 vs. L2	6.875	0.343; 13.407	0.313	0.039	0.062	1.9
Gait speed (m/s)	L4 vs. L1	0.152	0.024; 0.279	0.337	0.021	0.073	1.7
	**Age at diagnosis**						
Gait speed (m/s)	A2 vs. A1	−0.150	−0.266; −0.034	−0.333	0.012	0.131	1.5
	A3 vs. A2	0.179	0.046; 0.313	0.280	0.009	0.131	1.0
	**Immunosuppressant-refractory status**						
HGS (kg)	Yes vs. No	−5.018	−9.020; −1.017	−0.266	0.015	0.071	1.0
FFMI (kg/m^2^)	Yes vs. No	−1.214	−2.448; 0.019	−0.211	0.054	0.045	1.0
	**Line of biologic therapy**						
FMI (kg/m^2^)	Second-line vs. First-line IFX	2.618	0.461; 4.776	0.258	0.018	0.066	1.0
	**Laboratory**						
HGS (kg)	Hgb	0.418	0.291; 0.546	0.585	0.000	0.343	1.0
SMI (kg/m^2^)	Hgb	0.061	0.037; 0.085	0.491	0.000	0.241	1.0
FFMI (kg/m^2^)	FC	−0.001	−0.002; 0.000	−0.242	0.034	0.059	1.0

HGS—hand grip strength; SMI—skeletal muscle mass index; PBF—percent body fat; FFMI—fat free mass index; FMI—fat mass index; Hgb—hemoglobin; FC—fecal calcprotectin; IFX—infliximab.

**Table 6:** Multivariable linear regression—Dependent variable: HGS (kg).

Predictor	B	95% CI	Beta	*p*
Age	0.063	−0.091; 0.218	0.075	0.415
Sex	−11.100	−15.564; −6.636	−0.506	**<0.001**
BMI (kg/m^2^)	0.238	−0.091; 0.567	0.112	0.154
Disease duration (years)	−0.135	−0.357; 0.086	−0.109	0.227
Remission (active vs. remission)	−1.712	−4.794; 1.371	−0.086	0.272
Physical activity (MET total)	<0.001	0.000; 0.000	0.079	0.297
Immunosuppressant-refractory (yes vs. no)	−1.129	−3.963; 1.705	−0.060	0.430
Localization: L1 vs. L3	3.994	0.088; 7.900	0.164	**0.045**
Localization: L2 vs. L3	4.288	0.069; 8.507	0.166	**0.046**
Localization: L4 vs. L3	−1.339	−5.103; 2.425	−0.060	0.480
Hemoglobin (Hgb)	0.167	0.026; 0.309	0.234	**0.021**

R^2^ = 0.639; Adjusted R^2^ = 0.583; F (11,72) = 11.561; *p* < 0.001. Reference category for localization = L3 (ileocolonic). BMI—body mass index; MET—metabolic equivalent; CI—confidence interval. Bold *p*-values indicate statistical significance (*p* < 0.05).

**Table 7:** Multivariable linear regression—Dependent variable: SMI (kg/m^2^).

Predictor	B	95% CI	Beta	*p*
Age	0.002	−0.018; 0.023	0.016	0.819
Sex	−1.957	−2.538; −1.376	−0.510	**<0.001**
BMI (kg/m^2^)	0.226	0.183; 0.270	0.610	**<0.001**
Disease duration (years)	−0.014	−0.043; 0.015	−0.066	0.330
Physical activity (MET total)	<0.001	0.000; 0.000	0.017	0.761
Remission (active vs. remission)	−0.158	−0.565; 0.249	−0.045	0.443
Hemoglobin (Hgb)	0.008	−0.011; 0.026	0.060	0.424
Localization: L1 vs. L2	−0.722	−1.340; −0.105	−0.169	**0.023**
Localization: L3 vs. L2	−0.415	−0.970; 0.140	−0.126	0.141
Localization: L4 vs. L2	−0.708	−1.314; −0.102	−0.181	**0.023**

R^2^ = 0.791; Adjusted R^2^ = 0.762; F (10,73) = 27.553; *p* < 0.001. Reference category for localization—L2 (colonic). BMI—body mass index; MET—metabolic equivalent; CI—confidence interval. Bold *p*-values indicate statistical significance (*p* < 0.05).

**Table 8:** Multivariable linear regression—Dependent variable: Gait speed (m/s).

Predictor	B	95% CI	Beta	*p*
Age	0.001	−0.006; 0.007	0.033	0.859
Sex	−0.014	−0.113; 0.086	−0.031	0.786
BMI (kg/m^2^)	−0.001	−0.010; 0.009	−0.017	0.885
Disease duration (years)	0.003	−0.005; 0.010	0.102	0.522
Physical activity (MET total)	<0.001	0.000; 0.000	−0.016	0.886
Remission (active vs. remission)	−0.051	−0.144; 0.042	−0.126	0.280
Age at diagnosis: A1 vs. A2	0.127	−0.009; 0.264	0.228	0.067
Age at diagnosis: A3 vs. A2	0.204	0.022; 0.386	0.318	**0.029**
Localization: L2 vs. L1	0.075	−0.064; 0.215	0.145	0.283
Localization: L3 vs. L1	0.056	−0.059; 0.171	0.148	0.334
Localization: L4 vs. L1	0.137	0.007; 0.267	0.304	**0.040**

R^2^ = 0.209; Adjusted R^2^ = 0.088; F (11,72) = 1.729; *p* = 0.084. Reference categories: localization = L1 (ileal); age at diagnosis = A2 (17–40 years). BMI = body mass index; MET = metabolic equivalent; CI = confidence interval. Bold *p*-values indicate statistical significance (*p* < 0.05).

**Table 9:** Multivariable linear regression analyses for body composition parameters (PBF, FFMI, FMI).

Dependent Variable	Predictor	B	95% CI	Beta	*p*
PBF (%)	Age	−0.009	−0.130; 0.111	−0.011	0.878
	Sex	12.555	9.916; 15.195	0.582	**<0.001**
	BMI (kg/m^2^)	1.382	1.118; 1.645	0.663	**<0.001**
	Disease duration (years)	0.101	−0.076; 0.278	0.083	0.261
	Remission (active vs. remission)	0.269	−2.211; 2.748	0.014	0.830
	Physical activity (MET total)	<0.001	0.000; 0.000	−0.055	0.373
	Localization: L1 vs. L2	4.876	1.118; 8.635	0.203	**0.012**
	Localization: L3 vs. L2	3.682	0.307; 7.057	0.199	**0.033**
	Localization: L4 vs. L2	5.062	1.397; 8.727	0.230	**0.007**
PBF (%)—R^2^ = 0.750; F (9,74) = 24.627; *p* < 0.001					
FFMI (kg/m^2^)	Age	0.018	−0.025; 0.061	0.071	0.407
	Sex	−3.096	−4.030; −2.162	−0.490	**<0.001**
	BMI (kg/m^2^)	0.321	0.229; 0.413	0.509	**<0.001**
	Disease duration (years)	−0.085	−0.146; −0.025	−0.238	**0.007**
	Remission (active vs. remission)	−0.310	−1.189; 0.568	−0.053	0.483
	Physical activity (MET total)	<0.001	0.000; 0.000	−0.093	0.201
	Immunosuppressant-refractory (yes vs. no)	−0.496	−1.316; 0.324	−0.088	0.232
	Fecal calprotectin (FC)	−0.001	−0.002; 0.000	−0.183	**0.024**
FFMI (kg/m^2^)—R^2^ = 0.678; F (8,68) = 17.866; *p* < 0.001					
FMI (kg/m^2^)	Age	−0.002	−0.038; 0.035	−0.005	0.931
	Sex	3.154	2.375; 3.934	0.408	**<0.001**
	BMI (kg/m^2^)	0.631	0.553; 0.710	0.846	**<0.001**
	Disease duration (years)	0.059	0.005; 0.114	0.136	**0.034**
	Remission (active vs. remission)	0.604	−0.158; 1.366	0.086	0.119
	Physical activity (MET total)	<0.001	0.000; 0.000	0.064	0.215
	Line of therapy (2nd vs. 1st)	0.617	−0.465; 1.699	0.061	0.260
FMI (kg/m^2^)—R^2^ = 0.812; F (7,76) = 46.964; *p* < 0.001					

Reference categories: localization—L2 (colonic). BMI—body mass index; MET—metabolic equivalent; FC—fecal calprotectin; CI—confidence interval; PBF—percent body fat; FFMI—fat-free mass index; FMI—fat mass index. Bold *p*-values indicate statistical significance (*p* < 0.05).

## Data Availability

The data that support the findings of this study are available upon reasonable request from the corresponding author.

## Abbreviations

The following abbreviations are used in this manuscript:

HGS	Handgrip strength
BCP	Body composition parameters
SMM	Skeletal muscle mass
SMI	Skeletal muscle mass index
BFM	Body fat mass
PBF	Percent body fat
FFM	Fat-free mass
FFMI	Fat-free mass index
SARC-F	Strength, assistance with walking, rising from a chair, climbing stairs, and falls
IPAQ	International Physical Activity Questionnaire.
